# Protocol for the Implementation and Assessment of “MoodUP”: A Stepped Care Model Assisted by a Digital Platform to Accelerate Access to Mental Health Care for Cancer Patients Amid the COVID-19 Pandemic

**DOI:** 10.3390/ijerph18094629

**Published:** 2021-04-27

**Authors:** Diana Frasquilho, Ricardo Matias, Jaime Grácio, Berta Sousa, Fernando Luís-Ferreira, João Leal, Fátima Cardoso, Albino J. Oliveira-Maia

**Affiliations:** 1 Champalimaud Clinical Centre, Breast Unit, Champalimaud Centre for the Unknown, Champalimaud Foundation, 1400-038 Lisboa, Portugal; berta.sousa@fundacaochampalimaud.pt (B.S.); fatimacardoso@fundacaochampalimaud.pt (F.C.); 2 Champalimaud Research, Champalimaud Centre for the Unknown, Champalimaud Foundation, 1400-038 Lisboa, Portugal; 3 Champalimaud Research and Clinical Centre, Neuropsychiatry Unit, Champalimaud Centre for the Unknown, Champalimaud Foundation, 1400-038 Lisboa, Portugal; ricardo.matias@neuro.fchampalimaud.org (R.M.); jaime.gracio@research.fchampalimaud.org (J.G.); albino.maia@neuro.fchampalimaud.org (A.J.O.-M.); 4 Human Movement Analysis Lab, Escola Superior Saúde—Instituto Politécnico de Setuúbal, 2914-503 Setúbal, Portugal; 5 NOVA Medical School, NMS, Universidade Nova de Lisboa, 1169-056 Lisboa, Portugal; 6 Electrical Engineering Department, School of Science and Technology, CTS, FCT NOVA, Universidade Nova de Lisboa, 2829-516 Caparica, Portugal; flf@uninova.pt (F.L.-F.); jd.leal@campus.fct.unl.pt (J.L.)

**Keywords:** mental health, COVID-19 pandemic, anxiety, depression, oncology, collaborative healthcare

## Abstract

The COVID-19 pandemic has important consequences for the mental health of populations. Patients with cancer, already at risk for poor mental health outcomes, are not expected to be spared from these consequences, prompting the need for health services to improve responsiveness. This article presents the research protocol for an implementation study designed to describe the uptake of a well-studied and recognized system for the treatment of depression and anxiety (Stepped-care) during the specific context of a Pandemic in an oncological site. The system set-up will be assisted by a digital platform (MoodUP), where patients undergoing cancer treatment will be screened for anxiety and depressive symptoms, triaged by severity level and algorithm-matched to recommended interventions. Patients undergoing cancer treatment at a cancer clinic in Portugal will be invited to subscribe to the MoodUP platform where they will complete a self-reported questionnaire (Hospital Anxiety and Depression Scale) to screen their anxiety and depressive symptoms. Data will be instantly collected, and an algorithm will activate severity-matched intervention suggestions, through a case manager that will coordinate care. The specific objectives of this study will be to describe the implementation and acceptability of the care system by patients and staff, the barriers to and facilitators of implementation, the proportion of patients accessing the system and their pathways through the various stepped-care interventions, and patient perceptions regarding the feasibility and appropriateness of the eHealth platform. Moreover, exploratory analyses will be conducted to describe patterns of anxiety and depression symptoms variation across all patients, as well as within sociodemographically, clinically and contextually characterized subgroups, to characterize their care needs and access, as well as to explore for whom the MoodUP care system may be more appropriate. This study is expected to improve processes for collaborative mental healthcare in oncology and accelerate the digitalization of services, towards the improvement of mental healthcare access, and management of high-risk patients, during the COVID-19 pandemic.

## 1. Introduction

The COVID-19 pandemic is the most severe global health crisis of our recent history and has important effects on the mental health of populations [1,2,3,4]. While these effects have not yet been fully described and quantified, reports of mental distress are soaring, with the pandemic and social distancing measures reportedly contributing towards occurrence of low mood, stress, insomnia, anger, fear, loneliness and a sense of entrapment [2,4,5,6,7]. Also, the prevalence of clinically relevant anxiety and depression symptoms are likely to increase considerably compared to pre-COVID-19 pandemic levels [2,4,8]. Vulnerable populations for poor mental health include individuals with pre-existing physical and/or mental health conditions [2,4,5], including patients with cancer. Even before the COVID-19 Pandemic, patients with cancer already had significantly higher rates of depression and anxiety than the general population [9,10,11,12]. A systematic review and meta-analysis has suggested that depression and anxiety affect more than 20% and 10% of cancer patients, respectively, compared to 5% and 7% of the general population for past-year prevalence [10]. The fact that these patients are also at greater risk of becoming seriously ill or even dying of COVID-19 [11,12], may lead to disproportionate increases of fear and anxiety in this population. Consistently, findings from the early stages of the COVID-19 outbreak suggests a high prevalence of anxiety and depression symptoms in cancer patients [13,14]. Furthermore, it is likely that recurrent outbreaks of COVID-19 will be associated with intermittent social distancing measures [15,16], with inevitable economic consequences. Learning from our recent past, economic recession and the associated unemployment, income decline and debt, will be linked with further negative mental health outcomes and increased health inequalities [17,18,19].

Mental disorders are disabling and, if left undetected, even when mild, can progress in severity and become enduring. Common mental disorders (CMD’s), such as anxiety and depression, contribute for cumulative disadvantage in patients with cancer, entailing clinically relevant effects on quality of life, functionality, productivity and mortality [9,10,20,21,22,23,24,25]. Moreover, a recent study has shown that the annual health care use and costs in patients with cancer are 113% higher when depression is also present, naturally creating a significant impact on health systems [26]. Despite this evidence, it is consensual that, already before the pandemic, CMD’s are under-recognized and under-treated among patients with cancer, with mental health care delivered to fewer than 41% of those in need [9,27]. Possible barriers include inadequate screening, inability to tackle demand and/or insufficient collaborative care [28]. In Portugal, these may reflect conservative rates, since the country has one of the highest prevalence of CMD’s (23%) in Europe, representing the second largest cause of disability in the country, while access to mental health care is still below other European countries [29,30]. Furthermore, access to care is expected to decline due to the pandemic and its consequences on patients and services. In this context, mental healthcare service delivery needs to be re-designed to align with the physical distancing restrictions, while boosting its capacity. Efficient provision of mental health care within routine cancer care, optimizing health outcomes and quality of life, will be crucial to promote adaptation to the conditions of the pandemic and to face the crises ahead [9,23].

Collaborative care is a model of mental healthcare delivery with proven effectiveness in the general population, that may help tackle the mental care treatment gap in oncology [22,31]. This is a patient-centred mental healthcare model where multidisciplinary decision-making involves patients, case managers and health professionals, including, but not limited to, mental health professionals [22,31,32]. Within this model, the stepped-care strategy is considered optimal. It implies that evidence-based treatment follows successive levels, implemented and monitored systematically, according to patient needs [32]. The least resource-intensive treatment (e.g., self-help) is delivered first and care is stepped up if no significant health benefits are achieved [32]. Despite the growing needs, implementation of these models has not yet been adequately tested in the context of oncological care [22]. Moreover, in response to physical distancing, mental healthcare delivery must be complemented and expanded via digital health tools, further highlighting the need for urgent research on their real-world implementation and effectiveness [33].

This sets the rationale for this study, which intends to fill this knowledge and treatment gap by implementing and describing a collaborative stepped-care model to scale-up mental health care for cancer patients, amid the COVID-19 pandemic. An eHealth triage system platform (MoodUP) will be used to collect and monitor self-reported psychological distress (including symptoms of Depression and Anxiety), and to recommend interventions depending on individual needs, over time. The digital response complies with the social distancing measures to prevent COVID-19 spreading while ensuring access to existing and novel services [2]. Moreover, this innovative approach is expected to provide information and empowerment to better cope with anxiety and depressive symptoms. Thus, here we specifically aim to describe the implementation and acceptability of the care system by patients and staff, the barriers to and facilitators of implementation, the proportion of patients accessing the system and their pathways through the various stepped-care interventions, and to describe patient perceptions regarding the feasibility and appropriateness of the eHealth platform. Moreover, exploratory analyses will be conducted to describe patterns of variation of anxiety and depression symptoms across all patients, as well as within sociodemographically, clinically and contextually characterized subgroups, to describe their care needs and access, as well as to explore for whom the MoodUP care system may be more appropriate. We expect that, in addition to advancing and transferring scientific and technological knowledge on collaborative mental healthcare in oncology, this work will rapidly reorient care team to scaled-up and accelerated collaborative mental healthcare capacity, to improve management of high-risk patients.

## 2. Materials and Methods

### 2.1. Study Design and Setting

This observational operationalization study is designed to describe the uptake and the implementation of a well-studied and recognized stepped-care system, for the treatment of depression and anxiety in patients with cancer, during the specific context of a pandemic. Amid the COVID-19 pandemic, cancer patients reporting psychological distress will be referred by the oncology clinical team responsible for their care. Subsequently, with the assistance of MoodUP, a digital platform developed specifically for this study, patients will be screened and assessed for symptom severity, leading to the suggestion of severity-dependent treatment recommendations. The study will have a longitudinal design, using repeated measures of psychological distress to follow patients sharing a common experience, namely cancer and exposure to the pandemic context, across a 6 months period for each patient.

The study will be implemented at the Champalimaud Clinical Centre, where 6500 patients with cancer are treated, on average, per year. According to prevalence rates for CMD’s in Portugal (23%) [29] and based on the mentioned numbers, we expect 1500 of the patients to have CMD symptoms and only a fraction to be referred and accept to participate in the study. With conservative estimates of a 20% referral rate and 50% acceptance/participation rate, as well as a drop-out rate of 20%, we expect that 10 patients will complete the study per month. Participants will be selected based on their willingness to participate, using convenience sampling, for the total duration of the pandemic and the associated distancing restrictions with an expected reach number between 120 for 1 year duration and 240 for 2 years duration. The total number of participants will depend on the duration of the pandemic and the associated distancing restrictions, with a number between 120 (1 year) and 240 (2 years) expected. The end of the pandemic will be considered when, locally, COVID-19 incidence is low and government containment measures are no longer warranted.

Additionally, we will also invite patients and key stakeholders (health professionals from oncology and mental health, staff, study managers and administrators) from all care unit teams participating in the implementation of the MoodUP system, based on their availability to participate, to provide critical feedback on their testing experience via qualitative methods (e.g., online and/or phone individual interviews) to improve its overall design. Taking into consideration the differences in professional backgrounds among stakeholders, the number of participants will dependent on the number of interviews to be carried out until thematic saturation is reached, which is considered when no further concepts and themes are found and that may occur within 12 interviews [34].

### 2.2. Study Participants and Eligibility Criteria

Eligible participants will be adult patients (18 years or older) during and/or following cancer treatment, reporting feelings of psychological distress at any time during the COVID-19 pandemic. Participants must be capable of providing informed consent and have autonomous access to the internet through a mobile phone or computer, or access the internet via a patient nominated proxy. Patients will be excluded if they do not provide informed consent, are unable to read and/or understand Portuguese, have cognitive impairment (such as neurodegenerative disorder, dementia or psychosis) or cerebral metastases, require urgent psychiatric care and/or express suicidal ideation, are under active treatment for a mental disorder by a psychiatrist or psychologist, or ended treatment less than two months ago, have a history of psychosis or mania, or if they have an ongoing substance use disorder.

### 2.3. Participant-Selection and Consent Process

Patients will be identified by health professionals responsible for their oncology-related care, who will provide information about the MoodUP system and obtain patient informed consent. Patients will then be contacted by a MoodUP case manager (e.g., nurse), that will assist patients on how the system works and who will create personal accounts to access the online platform. Finally, patients will receive a confirmation link sent to the email address they provide, and by clicking the link will access a user account confirmation message granting access to MoodUP.

### 2.4. Ethics

The protocol was approved by the Champalimaud Foundation Ethics Committee which provides, in the light of the current legislation, the consent for processing personal data of patients. The research will comply with the Declaration of Helsinki and the corresponding ethical principles, as well as the applicable international, EU and national directive laws (EU Directive 2004/23/EC), in particular, the General Data Protection Regulation (EU GDPR) and national data protection laws. Written informed consent will be required for participation.

### 2.5. Outcome Assessment

Using participatory action research [35,36] approach, participants (patients) and key stakeholders (health professionals from oncology and mental health, staff, study managers and administrators) will be invited to give critical feedback and iteratively evaluate the MoodUP stepped care system via qualitative methods (e.g., using online and/or phone individual interviews online and/or phone interviews). The interviews will be used to explore what patients and key stakeholders think of the MoodUP methods and procedures, and also about the acceptability of a stepped care system for mental health in the oncological setting during the pandemic. We will follow an interview protocol similar to other relevant research in the area of health systems [37] and, as an example, the interviews may include questions for patients such as “What did you expect from treatment?”, “How well did the MoodUP solution helped you with the problems you wanted to work?” and “What, if anything, could have been done differently to improve your experience?”; and for key stakeholders such as “What are your thoughts about the MoodUP system?”, “How did you feel taking part of this solution for the patients?” and “What can be done to improve how the MoodUP system runs?”. The interviews will allow gathering data about the feasibility and appropriateness of the MoodUP platform and which procedures are efficient versus those that are perceived to be problematic and need improvement.

Moreover, anxiety and depression scores will be measured using the Hospital Anxiety and Depression Scale (HADS), which patients will complete online fortnightly [38,39,40,41]. This is a self-report questionnaire with 14 items, divided into subscales for anxiety for depression symptoms. It has shown good validity in web-based administration, is validated into Portuguese with good internal consistency (anxiety subscale Cronbach alpha of 0.76; depression subscale Cronbach alpha of 0.81), and is adequate for the screening of psychological distress in cancer patients [38,39,40,41]. Total HADS scores will be automatically computed and used to classify distress severity levels as ‘‘normal’’ (0–7), ‘‘mild’’ (8–10), ‘‘moderate’’ (11–14) and ‘‘severe’’ (15–21) [40,42].

### 2.6. Covariates

To adjust for possible differences in the experience of psychological distress, socio-demographic questions will also be asked and will include age, gender, education, employment status and occupation, perceived household income, marital status, family size and subjective social status [43]. Clinical variables will also be retrieved retrospectively from Personal Health Records (e.g., ICD-10 Classification; tumour biology, histological type, grade, previous/ongoing oncological treatments; Eastern Cooperative Oncology Group—ECOG—Performance status scale; genetic risk factors; prescribed medication and other chronic illnesses). Assessment of the proportion of participants who decline, drop-out and complete treatment will also be collected.

### 2.7. System of Delivering and Monitoring Mental Health

The MoodUP stepped care system builds on the knowledge of a multidisciplinary research team with strong know-how on oncology and mental health care, as well as eHealth technology, with ongoing participation in international projects for mental health monitoring in patients with cancer. The system will allow for screening and severity assessment of anxiety and depression symptoms, at baseline and across time, prompting treatment recommendations based on symptom severity. At baseline, patients will complete the HADS, that will also be used for follow-up assessments. All patients, irrespective of baseline distress score, will undergo active symptom monitoring every 2 weeks for 6 months follow-up, and HADS scores will be used to provide graphical feedback on severity to the patients [44,45]. After baseline assessment, a triage algorithm will alert the case manager via secure messaging about the HADS scores and symptom range and will recommend the level of intervention needed. The case manager coordinates care and makes sure the patient receives information on the recommended personalized intervention. Intervention is dynamic and varies over time depending on individual-level scores of distress and associated treatment needs. Nevertheless, patients reaching higher treatment levels will only step-down treatment levels at the discretion of their providers. Treatment recommendations will follow 4 intensity steps according to distress levels, as described in Table 1.

### 2.8. Technical and Service Description of the MoodUP Platform

**Figure 1:**
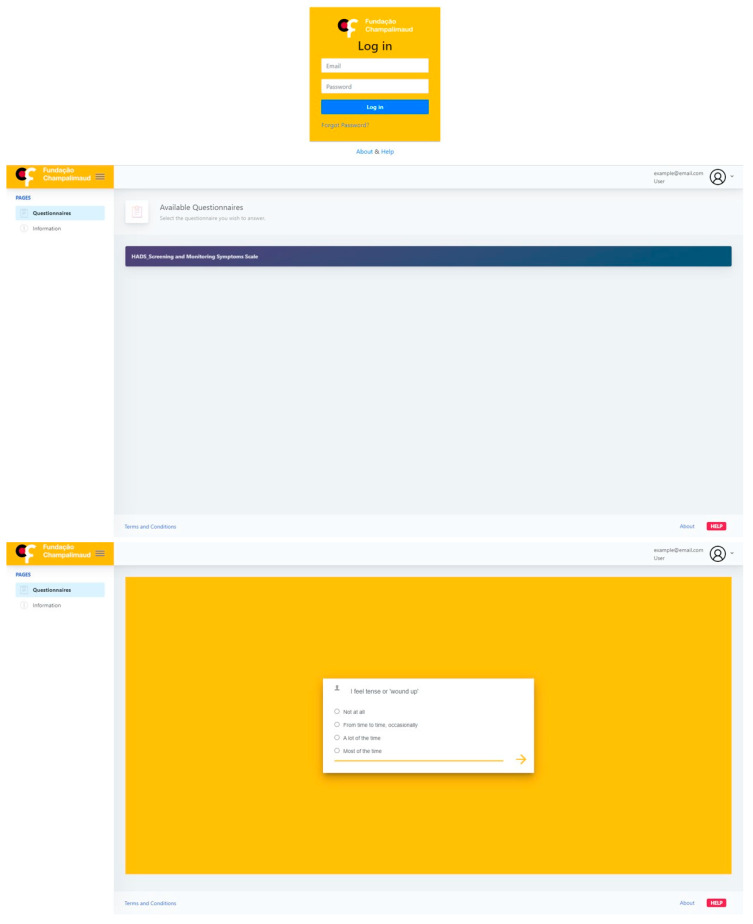
MoodUP platform prototype.

The MoodUP platform will be the main tool for integrating recruitment, data collection and delivery of the scientific results. It was developed and implemented locally, and a prototype is ready for operationalization (Figure 1).

Security is part of MoodUP platform architecture on all levels: software architecture, data centre architecture and network architecture. Regarding the data access level, data is encrypted when inserted at the database. Thus, in the case of a security breach, data would be unreadable and completely useless to safeguard privacy and data security. To ensure the security of the system, the architecture was designed so that the inner layer comprises the Champalimaud Foundation servers that are inaccessible from the outside WWW (internet). Both servers “Data MoodUP Server” and “Auth MoodUP Server” exist only in the inner network. Data is located in the “Data MoodUP Server” and all sensitive data is encrypted in the database, which is not directly accessible from the outside. But even in the remote possibility of a security breach, data is encrypted and the database is not accessible to unauthorized users. On the other side, the “Main MoodUP Server” allows access to data over “REST over HTTPS”, after request authorization to “Auth Server”, depending on the type of permission to each user described in Table 2.

The only connection allowed to “Auth and Data MoodUP Servers” is by the main server’s, hosted by the Champalimaud Foundation. Thus, the whole MoodUP system is behind the firewall and, the clients have only access to the Main MoodUP Server over the port 443 HTTPS and port 80 for HTTP, all other ports are closed. Patients and Case Managers will access the “MoodUP Server” via HTTPS, with ensured security by having this connection always encrypted. Also, the fragmentation of the system, enforces data protection for the data server from possible corruption in case of DOS (Distributed denial of service attack), since the Main Server can only make requests to the Data Server, after authenticating the user.

### 2.9. Planned Statistical Analyses

Due to the operational and observational nature of this study, we will essentially analyse qualitative data and secondarily we will conduct exploratory analysis using the HADS scale scores over time. Qualitative data on feasibility and appropriateness of the care system and eHealth platform, once transcribed, will be analysed and coded using an inductive approach and thematic analysis supported by QSR NVivo 10 software. Prominent patterns and concepts resulting from the analysis will be identified as themes. Quantitative and qualitative data may then be merged for improved knowledge around acceptability by patient categories. For instance, by their sociodemographic characteristics, psychological distress level, treatment adherence, tumour type and cancer treatment.

Furthermore, we will conduct exploratory analyses, including sociodemographics as frequency counts and percentages (with ranges where applicable), and continuous data from HADS scores as means and standard deviations. We will also use descriptive statistics to explore data about patient pathways using start and end points and whether their treatment was stepped up or down according to the HADS scores. Such analysis will be used to describe repeated measures of psychological distress, including anxiety and depression symptoms (compared to baseline HADS anxiety and depression scores), across all patients and within sociodemographically, clinically and contextually characterized groups, to understand for whom the system is more appropriate and beneficial. For inference with longitudinal data we will use linear mixed-effects models which can efficiently use all of the available data, allow for the characterization of the HADS responses over time while dealing with complex models for covariance and handling missing data without the need of imputation. The inclusion of covariates in the models will be informed by the relevant literature regarding potential confounding effects and univariate analysis. Moreover, we will also calculate the proportion of enrolled participants who adhered to the MoodUP system, and the dropout rates. To estimate the loss to follow up bias, we will compare the characteristics of the participants who drop-out of the study or are lost to follow up, to minimize the impact of such bias in the analyses. For all analyses power will be calculated post-hoc, given that the final sample number will be highly dependent on the duration of the pandemic. Data will be analysed using the SPSS statistical package, assuming type-I error probability (α) of 0.05.

## 3. Expected Results/Discussion

This project is expected to develop solutions for scaling-up of mental health care capacity to patients with cancer, while leveraging scientific and technological knowledge on the value of an eHealth collaborative mental health stepped care model in oncology. Furthermore, we expect to deliver a feasible and appropriate digital platform that can be independently adapted or expanded to different care settings, ensuring know-how transfer to other healthcare institutions, in order to optimize care at a distance. Notwithstanding, it is important to anticipate some difficulties that may arise in the implementation. Specifically, there are risks of not having a sufficiently powered sample due to the unknown referral rate, participation rate and drop-out rate during pandemic. Furthermore, even if power is sufficient, unforeseen problems in ongoing recruitment processes may lead to a final sample that may suffer from selection bias and is not representative of the patient population of interest.

## 4. Conclusions

This study lays the foundations for a collaborative stepped mental healthcare system assisted by a digital platform (MoodUP) that will be key for innovative mental health management among patients with cancer, focused on diagnostic and treatment solutions to improve their quality of life. This will incorporate improvements in the usual clinical practice, since the platform can provide real-time feedback to the health professionals and patients, and may help oncology and mental health teams improve coordination, liaison and reorientation, to scale-up and accelerate mental healthcare capacity for patient management amid COVID-19 pandemic, and for future crises.

## Figures and Tables

**Table 1:** Content of the MoodUP stepped care treatment recommendations.

Step 1. Absent/Nil symptoms—Watchful waiting.	This involves no intervention and is based on the principle of spontaneous recovery [28,32]. General information for dealing with psychological distress during the COVID-19 pandemic will be provided.
Step 2. Mild symptoms—Guided self-help [32]	Guided self-help based on the principles of cognitive behavioural therapy (CBT) will be provided [46]. Patients will engage in up to 5 brief weekly interactions (10 to 15 min) with a trained non-specialist member of the support team, via email or phone. This intervention will follow a guided self-help support manual based on international guidelines for remote Psychological First Aid (PFA) during the COVID-19 outbreak [47]. This is a well-established approach to assist people in distress, promoting healthy coping and feelings of safety, calming, and hope [48]. Patients will be asked to describe their current worries and problems, and to develop and implement a problem-solving plan. These interactions are not aimed at developing a patient-therapist relation but are only meant to give basic psychological support in working through the self-help method [28,48,49]. Moreover, the guided self-help support team members cannot prescribe pharmacological treatment or engage in psychotherapeutic intervention. The guided self-help manual guidelines allow the team members to assess whether the case needs a referral to higher levels of intervention intensity than initially proposed by the alert system.
Step 3. Moderate symptoms—Consultation psychiatry/psychology intervention	The case manager will contact the patient for a joint discussion of the current psychological distress and referral to the oncology staff. Oncology medical staff will be advised to follow treatment as usual, as they see fit. Collaborative online/telephone consultation with a psychiatrist and/or psychologist will be made available to the oncology staff for discussion of the range of interventions available.
Step 4. Severe symptoms—Fast-track to liaison psychiatry/psychology intervention	Immediate referral to liaison psychiatry or psychotherapy will be performed. Psychiatrist and/or Psychologist decision may be to refer to step down level of assistance (namely to guided self-help) or to step up the level of assistance to local or community/hospital for all patients that did not respond to any of the previous steps of treatment and require complex and specialized treatment.

**Table 2:** MoodUP platform type of users and privileges.

Users	Privileges
System Manager	Administration privileges such as creating, altering and deleting user accounts and altering or deleting data related to the questionnaires, but cannot access patient responses.
Patient	Exclusive access to an individual account and responding only to the questionnaires assigned by the health professionals.
Case Manager	Access to the administrative data of the patients, created by or assigned to her/him. Can also create questionnaires or assign questionnaires to a patient.
